# Single-Step Selection of Drug Resistant *Acinetobacter baylyi* ADP1 Mutants Reveals a Functional Redundancy in the Recruitment of Multidrug Efflux Systems

**DOI:** 10.1371/journal.pone.0056090

**Published:** 2013-02-07

**Authors:** Anthony J. Brzoska, Karl A. Hassan, Ellen J. de Leon, Ian T. Paulsen, Peter J. Lewis

**Affiliations:** 1 School of Biological Sciences, University of Sydney, Sydney, New South Wales, Australia; 2 Department of Chemistry and Biomolecular Sciences, Macquarie University, Sydney, New South Wales, Australia; 3 School of Environmental and Life Sciences, University of Newcastle, Callaghan, New South Wales, Australia; 4 Australian Institute for Bioengineering and Nanotechnology, University of Queensland, Brisbane, Queensland, Australia; University of Technology Sydney, Australia

## Abstract

Members of the genus *Acinetobacter* have been the focus recent attention due to both their clinical significance and application to molecular biology. The soil commensal bacterium *Acinetobacter baylyi* ADP1 has been proposed as a model system for molecular and genetic studies, whereas in a clinical environment, *Acinetobacter spp*. are of increasing importance due to their propensity to cause serious and intractable systemic infections. Clinically, a major factor in the success of *Acinetobacter spp*. as opportunistic pathogens can be attributed to their ability to rapidly evolve resistance to common antimicrobial compounds. Whole genome sequencing of clinical and environmental *Acinetobacter spp*. isolates has revealed the presence of numerous multidrug transporters within the core and accessory genomes, suggesting that efflux is an important host defense response in this genus. In this work, we used the drug-susceptible organism *A. baylyi* ADP1 as a model for studies into the evolution of efflux mediated resistance in genus *Acinetobacter*, due to the high level of conservation of efflux determinants across four diverse *Acinetobacter* strains, including clinical isolates. A single exposure of therapeutic concentrations of chloramphenicol to populations of *A. baylyi* ADP1 cells produced five individual colonies displaying multidrug resistance. The major facilitator superfamily pump *craA* was upregulated in one mutant strain, whereas the resistance nodulation division pump *adeJ* was upregulated in the remaining four. Within the *adeJ* upregulated population, two different levels of *adeJ* mRNA transcription were observed, suggesting at least three separate mutations were selected after single-step exposure to chloramphenicol. In the *craA* upregulated strain, a T to G substitution 12 nt upstream of the *craA* translation initiation codon was observed. Subsequent mRNA stability analyses using this strain revealed that the half-life of mutant *craA* mRNA was significantly greater than that of wild-type *craA* mRNA.

## Introduction

*Acinetobacter spp*. are ubiquitous environmental organisms, and are readily isolated from soil, water, human skin, food items, and sewage effluent. Over the past decade, *Acinetobacter spp*. have become the specific focus of increased research interest due to two disparate reasons. Firstly, the non-pathogenic soil-dwelling *Acinetobacter* species, *A. baylyi* ADP1, has been proposed as an alternative model for molecular, genetic, and metabolic studies, since this organism is naturally transformable, genetically malleable, and nutritionally diverse [1], [2], [3]. Laboratory studies using *A. baylyi* ADP1 are facilitated by the capacity of the organism to be transformed with exogenous DNA, in either linear or plasmid form, simply by incubating the DNA samples with logarithmic-phase *A. baylyi* ADP1 cells [2]. Moreover, integration of foreign DNA into the *A. baylyi* ADP1 chromosome occurs at high frequencies, without the need for extensive regions of homology to be present on transfected DNA molecules [1]. Secondly, despite occupying diverse environmental niches, most of the 31 described *Acinetobacter* genomospecies have the capacity to cause disease in humans [4]. *Acinetobacter spp*. have been estimated to cause up to 10% of all Gram-negative hospital infections and, alarmingly, may result in a mortality rate of between 30 and 75% [5]. In a clinical context, *Acinetbacter spp*. present a significant challenge since hospital strains which have emerged over the last decade have frequently acquired resistance to all practicable antimicrobial compounds, including drugs reserved for serious multidrug resistant infections [6]. Antimicrobial resistance in *Acinetobacter spp*. can be acquired *via* horizontal gene transfer (HGT) of preformed genes carried on plasmids, transposons, and integrons, or can be can be a result of the mutation of endogenous genes resulting in a resistance phenotype, as in the case of the upregulation of chromosomally encoded multidrug efflux systems [7]. To date, nine multidrug efflux systems have been characterised or partially characterised in *Acinetobacter spp*: RND transporters AdeABC, AdeDE, AdeIJK, and AdeFGH; MFS transporters AdeF/AmvA, CraA AedC and Tet(A); MATE transporter AbeM, and SMR transporter AbeS (reviewed in [8]). Transcriptional upregulation of RND transporters AdeABC, and AdeIJK results in increased antimicrobial resistance in clinical strains, while deletion or insertional inactivation of components of AdeABC, AdeDE, AdeIJK, AdeFGH, CraA, and AbeS has established a causal relationship between the presence of the transporter and high-level or intrinsic resistance of *Acinetobacter spp*. to one or more antimicrobial compounds [8].

In this work we used the non-pathogenic environmental *Acinetobacter* species *A. baylyi* ADP1 as a model for studies on the evolution of efflux-mediated antimicrobial resistance in the genus *Acinetobacter*. *A. baylyi* ADP1 offers several advantageous traits for studies on the genetic basis of resistance in *Acinetobacter spp*., including; (1) high level of conservation of ‘core’ genes (i.e., genes which are conserved across all *Acinetobacter* species) between *A. baylyi* ADP1 and other members of the *Acinetobacter* genus [9], [10], [11]; (2) high level of susceptibility of *A. baylyi* ADP1 to most commonly used chemotherapeutic agents [12]; (3) absence of strongly transcribed resistance gene determinants in *A. baylyi* ADP1 [12], enabling analysis of the development of resistance in an essentially resistance-free context; (4) natural transformability and recombinogenicity of *A. baylyi* ADP1 [2]; and (5) significant genome plasticity of *A. baylyi* ADP1 [2], [13], which is of particular importance in the evolution of antimicrobial resistance [14].

## Results

### Putative multidrug exporters encoded in *Acinetobacter* are commonly found within the core genome

**Figure 1:**
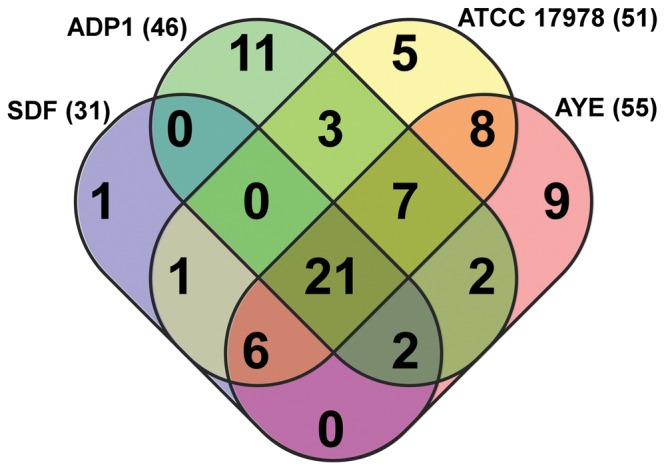
Venn diagram depicting conservation of putative drug efflux transporters encoded within representative sequenced strains of *Acinetobacter*. Each strain is represented by a colored oval, *A. baumannii* SDF (blue), AYE (red) and ATCC 17978 (yellow) and *A. baylyi* ADP1 (green). The number of efflux systems was determined using TransAAP. Numbers in brackets after strain names are the number of putative efflux systems encoded in that strain. Numbers in overlapping regions are the number of systems shared by those strains, as determined by comparative BlastP searches.

Multidrug exporters encoded within *Acinetobacter spp*. sequenced genomes were identified using the Transporter Automated Analysis Pipeline (TransAAP; www.membranetransport.org) [15]. The TransAAP uses a suite of tools to predict an organism's complete complement of membrane transport proteins from its predicted proteome [15]. Four diverse *Acinetobacter spp*. genomes were used for TransAAP multidrug transporter prediction: the soil commensal strain *A. baylyi* ADP1, clinical strains *A. baumannii* ATCC 17978 and *A. baumannii* AYE, and the human louse isolate *A. baumannii* SDF. Surprisingly, it was predicted that *A. baylyi* ADP1 encodes 46 multidrug transporters, almost as many as clinical strains *A. baumannii* ATCC 17978 (50), and *A. baumannii* AYE (55) (Fig. 1). The human louse isolate *A. baumannii* SDF, which is hypothesised to have undergone multiple rounds of genome reduction [10], encodes 31 putative multidrug transport proteins. Twenty-one multidrug transporters are predicted to be conserved across all four *Acinetobacter* genomes (Fig. 1).

### Evolution of Chloramphenicol Resistance in *A. baylyi* ADP1 Has Consequences for Bacterial Fitness

Single-step selection of *A. baylyi* ADP1 chloramphenicol resistant mutants was achieved by plating 2.08×10^8^ CFU of wild-type *A. baylyi* ADP1 cells onto Nutrient Agar (NA) containing either 10 or 20 µg/ml chloramphenicol. After 16 hours incubation at 37°C, three individual colonies were present on the plate containing 10 µg/ml chloramphenicol, and two on the plate containing 20 µg/ml chloramphenicol. Chloramphenicol resistant *A. baylyi* ADP1 strains selected on 10 µg/ml chloramphenicol were named Cm101, Cm102, and Cm103, and those selected on 20 µg/ml chloramphenicol were named Cm201 and Cm202 (Table 1).

**Table 1:** Bacterial strains, plasmids and primers.

Strain, plasmid or primer	Genotype or relevant characteristics^a^ ^b^	Source or reference
*Strains*		
*Acinetobacter baylyi* ADP1	wild-type (ATCC 33305)	(Juni and Janik, 1969)
Cm101	*A. baylyi* ADP1 efflux mutant selected on 10 µg/ml Cm	This study
Cm102	*A. baylyi* ADP1 efflux mutant selected on 10 µg/ml Cm	This study
Cm103	*A. baylyi* ADP1 efflux mutant selected on 10 µg/ml Cm	This study
Cm201	*A. baylyi* ADP1 efflux mutant selected on 20 µg/ml Cm	This study
Cm202	*A. baylyi* ADP1 efflux mutant selected on 20 µg/ml Cm	This study
ADP1Δ*adeJ*	*A. baylyi* ADP1 with *adeJ* deleted via allelic replacement. Kan^R^	This study
Cm101Δ*adeJ*	Cm101 with *adeJ* deleted via allelic replacement. Kan^R^	This study
Cm202Δ*adeJ*	Cm202 with *adeJ* deleted via allelic replacement. Kan^R^	This study
ADP1Δ*craA*	*A. baylyi* ADP1 with *craA* deleted via allelic replacement. Kan^R^	This study
Cm201Δ*craA*	Cm201 with *craA* deleted via allelic replacement. Kan^R^	This study
*Plasmids*		
pENTR/SD/D-TOPO®	2.6 kb Gateway Entry Vector; source of kanamycin resistance marker for gene knock-outs.	Invitrogen
*Primers*		
Kan-F	TAGCTGTTTCCTGGCAGCTC	
Kan-R	TCCCGTCAAGTCAGCGTAAT	
Kan-SCRN-R	GCCTGAGCGAGACGAAATAC	
AdeJ-FRONT-F	CGGTTACACGTACACCAACG	
AdeJ-FRONT-R	GAGCTGCCAGGAAACAGCTATCAGTCTACTCCTTATGCATGT	
AdeJ-BACK-F	ATTACGCTGACTTGACGGGATGCAAAAAGTATGGTCTATTTCAG	
AdeJ-BACK-R	CCAATTGTTGGAAGCTGGTT	
AdeJ-SCRN-F	TTGGCATTTTCTGATGCAAG	
CraA-FRONT-F	CTCTCGGCTTAAAATGTGGA	
CraA-FRONT-R	GAGCTGCCAGGAAACAGCTATTTGCATTGTGATCTACTAATCAA	
CraA-BACK-F	ATTACGCTGACTTGACGGGATCTGTTTGAAGCTCTGATATGTC	
CraA-BACK-R	ATCTCTGCTTTGGGGAGTCA	
CraA-SCRN-F	AAATGCAGCAATATGCAAAAGA	
AdeI_upF	GACGACGCATTTTGATGATG	
AdeI_upR	AAGCCTGATTCATCCACCAC	
AdeJ_upF	GCCGAGTTCGTTTTGAACAG	
AdeJ_upR	GCCACCAATGTTGAACACAC	
AdeK_upF	CGCGAGTCTATGCTGGAGTA	
AdeK_upR	CCAGCCTGTTCAGCATTTTT	
CraA_upF	GCATAACCGACCGCAGTAAT	
CraA_upR	ATAACGAGCCATGTCCTGCT	

a Cm: chloramphenicol.

b Kan^R^: kanamycin resistant.

**Figure 2:**
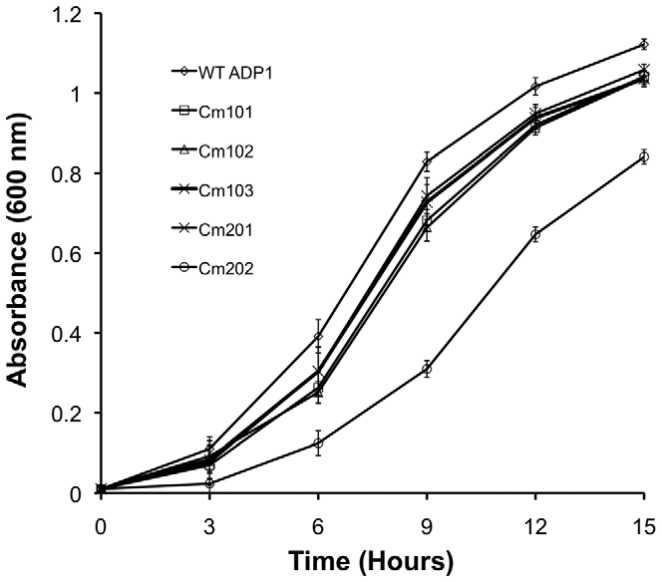
Growth characteristics of wild-type *A. baylyi* ADP1 or *A. baylyi* ADP1 single-step mutants over a time-course of 15 hours. Saturated overnight cultures of *A. baylyi* ADP1 strains were inoculated in fresh MH media to an OD_600_ of 0.01 in a volume of 200 µl prior to incubation at 37°C with shaking. Growth assays were conducted in duplicate using three biological replicates. Standard deviation is shown.

To assess the effect of acquired chloramphenicol resistance on bacterial fitness, we monitored the growth of mutant and wild-type *A. baylyi* ADP1 strains in rich medium (Meuller-Hinton; MH) over a time course of 15 hours (Fig. 2). Cm101, Cm102, Cm103 and Cm201 did not grow at a rate statistically significantly different to that of wild-type *A. baylyi* ADP1 (Table 2), despite having slightly longer doubling times in comparison to that of the wild-type strain. In contrast, strain Cm202 exhibited a significant growth defect compared to wild-type ADP1 (P-value 0.017; Table 2). Both the growth rate and the final cell density of Cm202 were markedly reduced compared to that of the wild-type strain, indicating that the development of chloramphenicol resistance in Cm202 was particularly deleterious to the fitness of the strain under non-selective conditions. Growth characteristics of strain Cm202 in comparison to other mutant strains suggested that mutations involved in the development of chloramphenicol resistance may differ between the single-step resistance mutants.

**Table 2:** Growth characteristics of wild-type and mutant *A. baylyi* strains.

Strain	Doubling time (m)^a^	P-value^b^
Wild-type ADP1	98.17±2.03	N/A
Cm101	109.88±9.58	NSD
Cm102	116.65±8.37	NSD
Cm103	112.06±3.85	NSD
Cm201	110.02±8.19	NSD
Cm202	149.72±9.71	0.017

a m: minutes. Standard Error is shown.

b Paired Student's t-test was used to determine if the exponential growth rates of mutant strains were significantly different than that of wild-type *A. baylyi* ADP1. A P-value below 0.025 indicates statistical significance. N/A: not applicable; NSD: no significant difference.

### Single-Step *A. baylyi* ADP1 Mutant Strains Exhibit Multidrug Resistance

To determine if the resistance spectrum of mutant strains extended beyond chloramphenicol, we conducted Minimal Inhibitory Concentration (MIC) analyses using a range of structurally and functionally diverse antimicrobial compounds. Multiple drug resistance phenotypes were observed for all single-step resistance mutants (Table 3). Such broad resistance profiles are often indicative of overexpression of multidrug efflux systems. The resistance phenotypes detected were not uniform between mutant strains, particularly between the resistance profile observed for strain Cm201 and the remainder of the resistant strains. Cm201 exhibits a relatively narrow multidrug resistance phenotype, showing two-fold increases in resistance to chloramphenicol, ethidium bromide and tetracycline. In comparison, Cm101, Cm102, Cm103 and Cm202 exhibit increased resistance levels to a much wider spectrum of drugs, albeit with differences in resistance levels between some strains, e.g., Cm202 shows a two-fold higher MIC for chloramphenicol, norfloxacin and tetracycline when compared to that of the other mutant strains. This marked difference in the efflux profile of Cm201 compared to that of the other single-step mutant strains suggested that a different transporter(s) was responsible for the substrate specificity exhibited by this strain. None of the mutant strains exhibited MICs greater than that of wild-type *A. baylyi* ADP1 for colistin, DAPI, kanamycin or amikacin (data not shown).

**Table 3:** MIC of compounds tested against *A. baylyi* ADPI strains.

	MIC (µg/ml)^a^ ^b^							
Strain	Cm	Et	Bc	Ch	SDS	Nor	Tc	Tm
**Wild-type ADP1**	6	16	5	2.5	400	1.024	0.1875	22.5
**Cm101**	12 (2)	128 (8)	10 (2)	5 (2)	800 (2)	4.096 (4)	0.75 (4)	90 (4)
**Cm102**	12 (2)	128 (8)	10 (2)	5 (2)	800 (2)	4.096 (4)	0.75 (4)	90 (4)
**Cm103**	12 (2)	128 (8)	10 (2)	5 (2)	800 (2)	4.096 (4)	0.75 (4)	90 (4)
**Cm201**	12 (2)	32 (2)	5 (0)	2.5 (0)	400 (0)	1.024 (0)	0.375 (2)	22.5 (0)
**Cm202**	24 (4)	128 (8)	10 (2)	5 (2)	800 (2)	8.192 (8)	1.5 (8)	90 (4)
**ADP1Δ** ***adeJ***	1.5 (−4)	4 (−4)	2.5 (−2)	1.25 (−2)	25 (−16)	0.064 (−16)	0.046875 (−4)	5.625 (−4)
**Cm101Δ** ***adeJ***	1.5 (−4)	4 (−4)	1.25 (−4)	0.625 (−4)	25 (−16)	0.064 (−16)	0.046875 (−4)	5.625 (−4)
**Cm202Δ** ***adeJ***	1.5 (−4)	4 (−4)	1.25 (−4)	0.625 (−4)	25 (−16)	0.064 (−16)	0.046875 (−4)	5.625 (−4)
**ADP1Δ** ***craA***	1.5 (−4)	8 (−2)	5 (0)	2.5 (0)	400 (0)	1.024 (0)	0.1875 (0)	22.5 (0)
**Cm201Δ** ***craA***	1.5 (−4)	8 (−2)	5 (0)	2.5 (0)	400 (0)	1.024 (0)	0.1875 (0)	22.5 (0)

a Fold change compared to wild-type MIC is given in brackets.

b Cm: chloramphenicol; Et: Ethidium bromide; Bc: Benzalkonium chloride; Ch: Chlorhexidine; SDS: Sodium Dodecyl Sulfate; Nor: Norfloxacin; Tc: Tetracycline; Tm: Trimenthoprim.

### Multidrug Resistance in Single-Step Mutants is a Result of Active Efflux

**Figure 3:**
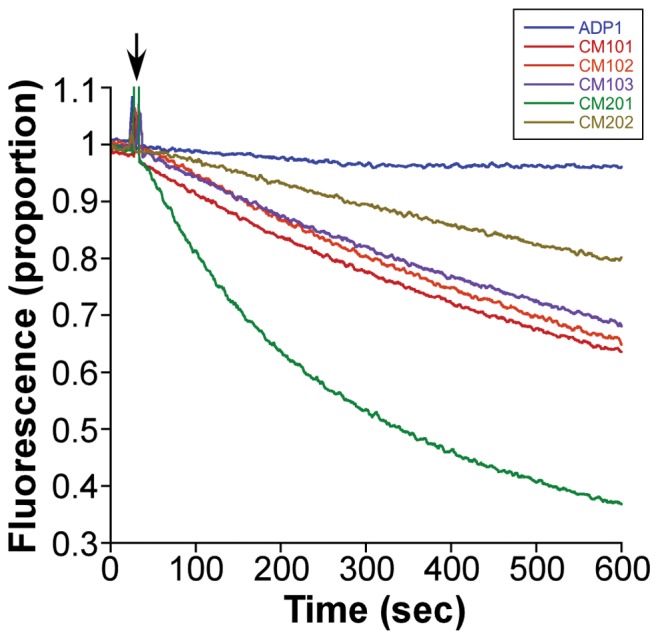
Efflux of ethidium bromide by *A. baylyi* ADP1 cells. Wild-type and mutant strains were loaded with 15 µM ethidium bromide in the presence of the protonophore CCCP. Cells were energised using 0.4% glucose at the point indicated by an arrow to initiate efflux. Efflux was monitored fluorometrically using an excitation wavelength of 530 nm and emission wavelength of 610 nm. Assays were conducted in duplicate, and a representative experiment is shown.

Each of the mutant strains exhibited an increased level of resistance to ethidium bromide when compared to the wild-type strain. Therefore, to confirm efflux as the mechanism of resistance in the single-step mutants, we conducted fluorimetric ethidium bromide transport assays. Each of the mutant strains was shown to actively export ethidium bromide at a rate significantly greater than the wild-type strain (Fig. 3). Of note, the rate of ethidium bromide efflux was not uniform across all of the mutant strains, further strengthening the hypothesis that different exporters were responsible for the resistance phenotypes observed in the different mutant strains. Interestingly, the initial rate of ethidium bromide efflux from strain Cm201 was far greater than that from the other single-step mutants, despite this strain displaying a lower overall ethidium bromide resistance level.

### Multidrug Resistant *A. baylyi* Mutants Exhibit Upregulation of Different Efflux Pumps

To identify multidrug efflux pumps involved in resistance phenotypes observed for mutant strains, we performed quantitative real-time PCR (qRT-PCR). Data generated using TransAAP (see above) was used to design oligonucleotides to all 46 multidrug efflux pumps predicted to be encoded within the *A. baylyi* ADP1 chromosome. These primer sets were then employed in a high-throughput, qRT-PCR-based genome-wide survey of multidrug efflux pump transcription. Basic data generated in this survey was used to identify transporters which were strongly upregulated (>5 fold), and these transporters were subjected to further qRT-PCR-based analysis. Preliminary qRT-PCR analyses determined that in each of the chloramphenicol resistant strains, only one putative multidrug efflux system was significantly upregulated (data not shown). As predicted from drug resistance and ethidium bromide transport phenotypes, qRT-PCR outputs indicated that not all single-step selected multidrug resistant *A. baylyi* ADP1 strains exhibit upregulation of the same efflux determinant (Table 4). Strains Cm101, Cm102, Cm103 and Cm202 showed upregulation of the gene ACIAD2944, whereas in Cm201 ACIAD3292 was upregulated. The qRT-PCR analyses also revealed differences in the level of upregulation of the efflux pump transcripts between the mutant strains (Table 4). Strains Cm101, Cm102, Cm103 (selected on 10 µg/ml chloramphenicol) show over 5-fold upregulation of ACIAD2944, whereas strain Cm202 (selected on 20 µg/ml chloramphenicol) shows nearly an 8-fold upregulation of the same gene. This increased upregulation of ACIAD2944 in Cm202 compared to the other mutant strains may account for its reduced fitness (Fig. 2). In contrast, Cm201 shows upregulation of ACIAD3292 at a level 34.4 fold higher that wild-type ADP1, with limited observed deleterious effects to bacterial growth.

**Table 4:** Fold changes in expression of multidrug efflux systems in the chloramphenicol resistant mutant strains as determined by qRT-PCR.

Strain	Gene Upregulated^a^	Fold Change^b^
Cm101	ACIAD2944	5.28±0.47
Cm102	ACIAD2944	5.23±0.37
Cm103	ACIAD2944	5.59±0.55
Cm201	ACIAD3292	34.44±2.69
Cm202	ACIAD2944	7.70±0.35

a Locus tag of *A. baylyi* ADP1 upregulated transporter.

b Standard deviation is shown.

The expression levels of the 46 putative efflux systems of ADP1 was determined by qRT-PCR to identify those that were upregulated in the mutant strains. Those showing greater than 5 fold-upregulation are included in the table.

### Mutant Strains Exhibit Upregulation of Functionally Distinct Transporters: RND *adeJ* or MFS *craA*

**Figure 4:**
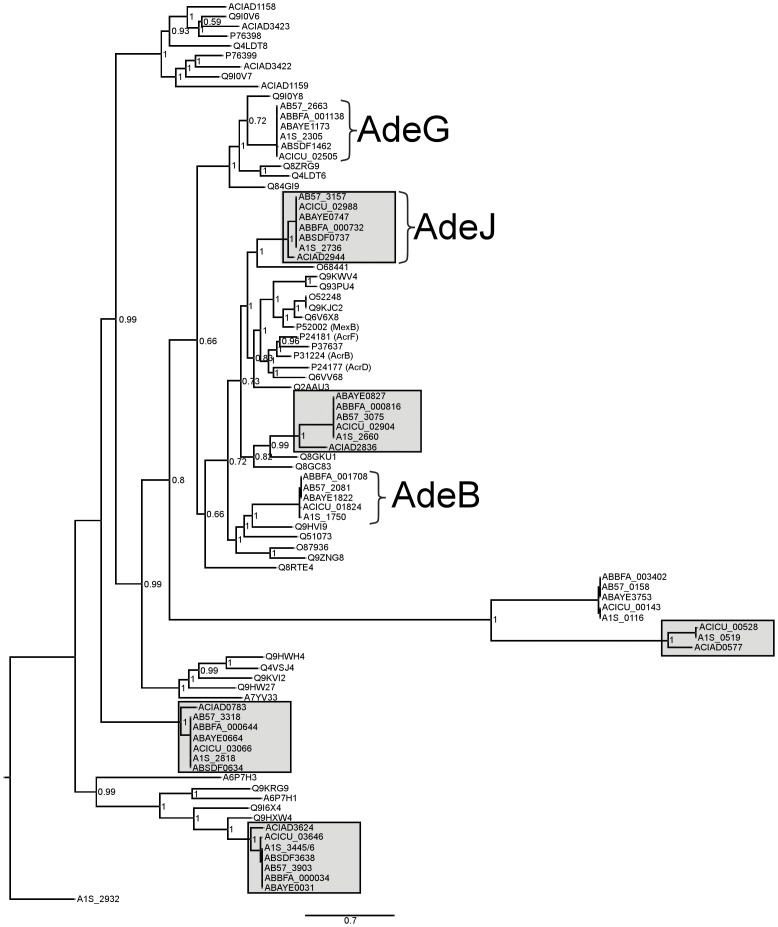
Bayesian tree depicting phylogenetic relationships of putative drug exporting RND superfamily transporters identified within the genomes of sequenced *Acinetobacter* spp. (as determined using TransAAP). Also included are previously characterized transporters classified within the Hydrophobe/Amphiphile Efflux-1 family of the RND superfamily, as listed in the Transporter Classification Database (www.tcdb.org). The protein sequence of the *A. baumannii* ATCC 17978 RND efflux transporter from the Heavy Metal Efflux family, CzcA (A1S_2932), was used as an out-group. Branch lengths are proportional to phylogenetic distance and the confidence of each node is indicated by posterior probabilities. Proteins othologous to the major RND drug efflux systems encoded in Acinetobacter, AdeB, AdeJ and AdeG are indicated. Gray shaded boxes denote clades that include orthologs from both *A. baumannii* and *A. baylyi*.

BLASTP searches revealed that ACIAD2944 exhibits 89% identity to the *A. baumannii* RND pump AdeJ [16], whilst ACIAD3292 exhibits 80% identity to the *A. baumannii* DHA1 MFS pump CraA [17]. The upregulation of these different classes of efflux pumps is of particular note, since this phenomenon illustrates a functional redundancy involved in the evolution of exporter-mediated resistance in *Acinetobacter spp*. in response to challenge with inhibitory concentrations of antibiotics. To examine the relatedness of the proteins encoded by ACIAD2944 and ACIAD3292 with a broad range of other RND and DHA1 MFS transporters (within the TCBD; www.tcdb.org), we conducted pairwise similarity analyses (Datasets S1 and S2). These analyses indicated that ACIAD2944 and ACIAD3292 are close orthologs of their *A. baumanni* AdeJ and CraA counterparts, since they share significantly higher levels of similarity with these proteins than with other RND or MFS transporters, respectively (Datasets S1 and S2). Further support for this observation is apparent in phylogenetic analyses of the proteins, i.e., the majority of drug exporting RND proteins encoded in *A. baumannii* share a close ortholog in *A. baylyi* ADP1 (Fig. 4).

### Deletion of *adeJ* or *craA* Induces Antimicrobial-Hypersusceptibility in *A. baylyi* ADP1

In order to confirm that the AdeJ (ACIAD2944) or CraA (ACIAD3292) transport proteins are independently responsible for the observed multidrug resistance phenotypes in single-step mutants, we deleted the *adeJ* or *craA* genes in both the drug resistant and wild-type *A. baylyi* ADP1 strains (Table 1), and conducted MIC assays on the resultant deletion derivatives. *adeJ* was not deleted in Cm102 and Cm103, since these strains exhibit an identical multidrug resistance profile to Cm101.

The deletion of either *adeJ* or *craA* resulted in the complete abrogation of resistance phenotypes observed in the respective mutant strain backgrounds (Table 3), indicating that the upregulation of the respective transporters was solely responsible for the resistance phenotypes observed. Moreover, and of particular note, we observed both *adeJ* and *craA* deletion derivatives showed hyper-susceptibility phenotypes for a range of compounds tested (Table 3). *adeJ* knockout strains show a reduction in MIC values of at least two fold (and up to 16 fold) in comparison to wild-type against all compounds tested. In contrast, *craA* deletion derivatives showed a decreased MIC for chloramphenicol and ethidium bromide only. Upregulation of *craA* transcription resulted in a modest increase in the MIC for tetracycline, however, the deletion of this gene does not result in a hyper-susceptibility phenotype for this compound. Hyper-susceptibility phenotypes observed here suggest that both *adeJ* and *craA* transport complexes play an important role in the homeostasis of toxic compound efflux in *Acinetobacter spp*.

To determine if the growth rates of *A. baylyi* strains in which either *adeJ* or *craA* were deleted were different to that of the wild-type strain, we conducted bacterial growth assays, essentially as described above. Interestingly, statistical analysis of this growth data showed that the deletion of *adeJ* in Cm202 (Cm202Δ*adeJ*) ameliorated the growth defect which was previously observed for this strain (data not shown). This result suggests that the ∼8-fold upregulation of *adeJ* in Cm202 is responsible for the observed differences in the growth rate of this strain in comparison to wild-type cells. The growth rates of wild-type *A. baylyi* ADP1 or other mutant strains in which efflux pumps had been deleted were not statistically different to that of the wild-type strain.

### Sequencing of Putative Transporter Regulatory Regions Reveals Differences in the Basis for *adeJ* and *craA* Upregulation

In an effort to determine the basis for the upreglation of efflux pump mRNA transcription in *A. baylyi* ADP1 single-step mutants, we sequenced regions upstream of structural genes encoding the AdeIJK and CraA efflux pump modules. This sequencing strategy would enable mutations potentially affecting promoters or regulatory motifs of the core pump genes to be identified. Genes encoding the *A. baylyi* ADP1 AdeIJK proteins reside in a region which is syntenous to the *adeIJK* operon in *A. baumannii* AB0057 ([16], data not shown), suggesting it is also an operon. Nevertheless, we sequenced across intergenic regions both upstream of and within the *adeIJK* operon, in single-step mutants which upregulate the transcription of *adeJ*. No mutations were found, indicating that a potential mutation elsewhere in the genome, possibly affecting expression of a global transcriptional regulator, may be responsible for the derepression of *adeIJK*. Similarly, *A. baumannii* AB0057 single-step mutants which exhibit upregulated transcription of *adeJ* [18] did not exhibit mutations within the putative *adeIJK* operator region, highlighting similarities between this *A. baumannii* strain and *A. baylyi* ADP1. However, it was recently shown that that a TetR-like regulator, AdeN, is responsible for the repression of the *adeIJK* operon in *A. baumannii*, and that mutations within the *adeN* structural gene results in the deprepression of *adeJ* in single-step mutants selected *in vitro* [19]. Therefore, considering the close phylogenetic similarities that *A. baylyi* ADP1 *adeJ* shares with its *A. baumannii* counterpart (Fig. 4), it is possible that the regulatory mechanisms which govern the repression of the *adeIJK* operon in *A. baylyi* ADP1 share mechanistic similarities with those recently described for *A. baumannii*. The role of putative AdeN homologues in the regulation of *adeIJK* in *A. baylyi* ADP1 warrants further investigation.

In contrast to the tripartite RND system AdeIJK, the CraA MFS transporter is encoded by a single gene, which appears not to reside in a bi- or multi-cistronic operon on the *A. baylyi* ADP1 chromosome. We sequenced a 486 bp region immediately upstream of the *craA* ORF in order to identify mutations which may affect the *craA* promoter or, potentially, an operator region of the gene, in the *craA* upregulated strain, Cm201. Interestingly, this sequencing strategy revealed a T to G substitution 12 base-pairs upstream from the *craA* translation initiation codon. The contribution of this mutation to multidrug resistance is intriguing, since it does not appear to coincide with a potential *craA* transcriptional promoter. A putative *craA* ribosome binding site (RBS), AGTAG, can be observed seven base pairs upstream from the *craA* translation initiation site, and resides immediately adjacent to the mutated residue. However, the observed substitution does not appear to extend or improve the putative RBS sequence. No known regulatory motifs are present within the vicinity of the mutation, although the mechanisms of transcriptional regulation of the *craA* promoter, if any, remain to be elucidated. The close proximity of the mutation to the putative RBS suggests that that the mutated residue forms part of the 5′ untranslated region (UTR) of the mature mRNA transcript.

### T to G Substitution in *craA* 5′ Upstream Region Increases mRNA Transcript Stability

**Figure 5:**
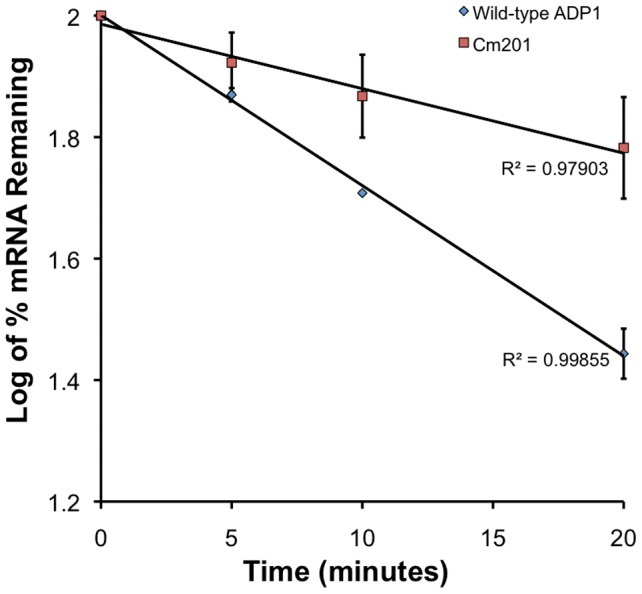
Stability of *craA* mRNA transcript in wild-type and Cm201 backgrounds. Wild-type and Cm201 cells were grown to mid-logarithmic phase before transcription was arrested with the addition of 200 µg/ml rifampicin. Culture samples were harvested at time points T = 0, 5, 10 and 20, before RNA was extracted and cDNA was synthesised. qRT-PCR was conducted in duplicate using two biological replicates, and the abundance of the *craA* transcript relative to the *rpoB* transcript in transcriptionally arrested cells was determined. R^2^ values and standard deviation is shown.

Mutations in the 5′ UTR of efflux pump transcripts have been shown induce multidrug resistance in *Staphylococcus aureus* [20], *Bacillus subtilis* [21] and in *Neiserria gonorrhoeae* [22]. The presence of a mutation in the 5′ UTR of the gene transcripts of *S. aureus norA*, *B. subtilis bmr3* and *N. gonorrhoeae mtrC-mtrD-mtrE* correlates with an increase in the half-life of the exporter gene mRNA. Despite the fact these stabilising mutations lie 80–120 nt upstream of the translation start site, and the mutation in Cm201 is only 12 nt upstream of the translation start site, we investigated the stability of *craA* mRNA in wild-type *A. baylyi* ADP1 and Cm201 cells using qRT-PCR. These assays revealed that wild-type *craA* mRNA transcripts decayed much more rapidly than that of the mutant mRNA transcripts (Fig. 5), indicating that the T to G substitution 12 base pairs distal to translational start point of *craA* enhances efflux pump transcript stability. The extrapolated wild-type *craA* mRNA half-life was 24 min, whereas it was nearly 3-fold longer at 65 min in Cm201. It is unknown at this stage how this mutation in the 5′ UTR of the *craA* transcript contributes to mRNA stability.

## Discussion

Infections involving *Acinetobacter spp*. are often difficult to treat, owing to the organism's propensity to rapidly and efficiently acquire resistance genes from the environment, or by exploiting their intrinsic resistance potential by overexpressing core resistance factors, as observed in these investigations. Recent clinical isolates of *Acinetobacter baumannii* have shown resistance to all useful chemotherapeutic compounds, leading researchers to term this state of intractability ‘pan-drug’ resistance [4].

Recently, the non-pathogenic soil-dwelling *Acinetobacter* species *A. baylyi* ADP1 has been proposed as a novel model organism for metabolic and genetic studies, since, amongst other traits, *A. baylyi* ADP1 is highly recombinogenic and easily transformable. Initially, we conducted a bioinformatic survey in order to determine the conservation of multidrug efflux pumps across four divergent species of the genus *Acinetobacter*, allowing us to determine the general utility of *A. baylyi* ADP1 for the study of multidrug efflux. Our bioinformatic survey indicated that more than 75% of the putative efflux systems encoded in *A. baylyi* ADP1 were shared with at least one of the *A. baumannii* strains, and approximately half (21 of 46) of these systems were shared with all other *Acinetobacter* species analysed (Fig. 1). Of particular note, *A. baylyi* ADP1 shares approximately 70 and 67% of its core genome efflux determinants with clinical strains AYE and ATCC 17978 respectively. The high level of conservation of efflux determinants between *A. baylyi* ADP1 and the two clinical isolates was somewhat surprising, since the accumulation of newer pre-formed efflux genes from other closely related species in the hospital environment is a predominant factor in the success of *A. baumannii* as a clinically important species, and as such, it could be expected that the types of pumps encoded by clinical species may differ significantly from that of a primarily soil-dwelling organism.

In order to study the efflux response of *A. baylyi* ADP1 to antimicrobial compounds, we adapted wild-type cells to 10 or 20 µg/ml chloramphenicol *via* a single-exposure selection assay. Chloramphenicol was chosen for this assay since many clinical *A. baumannii* strains exhibit intrinsic resistance to this drug [23], and the basis of this resistance is likely to be due to antimicrobial efflux, or the alteration of membrane permeability [23]. Results of our single-step selection assay showed that the reservoir of chloramphenicol resistant cells in populations of *A. baylyi* ADP1 is relatively low, with only approximately 1 in every 100 million cells showing resistance to chloramphenicol at or above the concentrations used. However, the resistance phenotype exhibited by these selected strains is indicative of an underlying presence of mutant cells in *Acinetobacter spp*. populations, even in the absence of selection. Bacterial growth assays using single-step chloramphenicol mutants surprisingly showed that four of the five strains (Cm101, Cm102, Cm103 and Cm201) adapted to tolerate higher concentrations of chloramphenicol did not show a statistically significant difference in growth rates compared to wild-type *A. baylyi* ADP1 (Table 2). Cm201 did not exhibit a growth defect despite its upregluated efflux determinant, *craA*, showing a ∼34 fold increase in transcription compared to wild-type *A. baylyi* ADP1 cells (Table 4). On the other hand, the ∼8 fold upregulation of *adeJ* in Cm202 resulted in a significant growth defect (Fig. 2). The upregulation of *adeJ* at lower levels (strains Cm101, Cm102, Cm103; ∼5.5 fold) did not result in a significant negative impact to bacterial fitness, indicating that the ∼5.5 fold upregulation of *adeJ* may be approaching the upper limit for the transcription of this gene without serious physiological consequences to the host. Corroborating this observation, bacterial growth assays showed that Cm202Δ*adeJ* grew at a rate which was not statistically different to that of wild-type *A. baylyi* ADP1 cells (data not shown), indicating that the ∼8-fold upregulation of *adeJ* was responsible for the deleterious effects observed. Previous studies have shown that the overexpression of the AdeIJK complex in both *A. baumannii* and *E. coli* is toxic to the host [16], and multidrug resistant *A. baumannii* strains, either isolated clinically or selected *in vitro*, have shown *adeJ* to be upregulated to an upper limit of approximately 5–6 fold [18], [24]. Our observations, in concert with those reported by Rajamohan *et al.* [24], Coyne *et al.*, [18], and Damier-Piolle *et al.* [16] indicate that environmental and evolutionary pressures are unlikely to select for *Acinetobacter spp*. which harbour mutations which strongly upregulate the transcription of *adeJ*, possibly due to the detrimental impact on overall cell fitness.

MIC analyses showed strains in which *adeJ* was upregulated were resistant to a wider range of antiseptics and disinfectants than Cm201, in which *craA* was upregulated. These data largely corroborate previous results describing substrate recognition profiles for both transporters [16]; *adeJ*, and [17]; *craA*, however, one key difference in substrate specificity can be noted. Damier-Piolle *et al.* [16] reported that ethidium bromide could not be transported by the AdeIJK system in *A. baumannii*, whereas in our study, *A. baylyi* ADP1 *adeJ* mutant strains show both an increase in ethidium bromide MIC (Table 3) and in the active transport of the compound (Fig. 3).With respect to CraA, our observations for the strong substrate specificity towards chloramphenicol of this transporter were in accordance with those reported by Roca *et al.* [17]. However, in this work we determined that ethidium bromide is also a substrate of this efflux protein, demonstrating that CraA is a multidrug efflux system.

It is noteworthy that the MIC for ethidium bromide in the *craA* upregulated strain Cm201 was much lower than that of the *adeJ* mutant strains, despite the high rate of ethidium bromide efflux observed for this strain. However, the transport experiments show only the initial rate of ethidium efflux from cells pre-loaded with a sub-MIC concentration of ethidium, and may not directly reflect the final resistance level of the cells. Cells overexpressing the AdeIJK efflux system may, for a number of reasons, be able to maintain a higher ethidium concentration gradient across their membranes than cells overexpressing CraA, despite the apparently slower initial rate of efflux. Most importantly, the AdeIJK tripartite complex spans both the inner and outer membranes of *Acinetobacter* cells, and facilitates substrate transport into the extracellular matrix [25], whereas, CraA is likely to move substrates into the periplasmic space. Over time the substrate that has accumulated in the periplasm may more readily re-enter the cytoplasm by diffusion than the substrates transported into the extracellular matrix by the AdeIJK system [27].

Differences in the initial ethidium efflux rates may also be related to the location of substrate capture by the respective transport systems. MFS transporters, such as CraA, are likely to capture their substrates from within the cytoplasm or inner-leaflet of the inner-membrane, whereas RND efflux systems, such as AdeIJK, may exclusively, or at least primarily, capture substrates from within the periplasmic space and not from the cytosol [26], [27]. Therefore, in cells over-expressing *adeIJK*, the movement of ethidium out of the cytosol is still reliant on either passive diffusion or on the action of simple transporters, which were not expressed above background levels in the *adeIJK* over-expression strains.

We sought to determine the genetic basis for the upregulation of *adeJ* and *craA* in the mutant strains. From the results of transcription analyses (Table 4), we speculated that at least three different mutations are present within the selected *A. baylyi* ADP1 single-step mutants, since 1) Cm201 upregulates a different transporter compared to the other selected strains and 2) Cm202 upregulates *adeJ* to a higher level than the other strains in which *adeJ* is upregulated. However, sequencing the promoter and intergenic regions in *adeJ* upregulated strains did not reveal any mutations, suggesting that a mutation in a global regulator of the AdeIJK efflux pump may be responsible for the upregulation of transcription in these strains. Indeed, a recent report describes a TetR-like regulator, AdeN, which is responsible for the regulation of *adeIJK* in *A. baumannii* [19]. The role of AdeN in the regulation of *adeIJK* in *A. baylyi* ADP1 remains to be determined, and warrants further investigation. However, if a homologue of AdeN does indeed regulate *adeIJK* in *A. baylyi* ADP1, mutations arising in *adeN* in Cm202 may differ from those affecting Cm101, Cm102, and Cm103, since *adeJ* in Cm202 is upregulated to a higher level compared that of the other strains in which *adeJ* is upregulated. Alternatively, *adeIJK* may be subject to more than one level of regulation in *A. baylyi* ADP1, thus accounting for the higher level of *adeJ* transcription observed in Cm202.

In contrast, sequencing the region upstream from the *craA* ORF revealed a T to G substitution 12 basepairs upstream from the predicted *craA* initiation codon. A putative *craA* RBS, AGTAG, can be observed in this region, but the upstream mutation does not appear to extend or improve the *craA* RBS. In light of other work which describes mRNA stabilising mutations in the 5′ UTR of efflux transcripts [20], [21], [22], we explored the ability of the T to G substitution in the putative *craA* transcript to increase the *craA* mRNA half-life. Using a qRT-PCR based assay, the *craA* mutant transcript was determined to be approximately 2.7 fold more stable than *craA* mRNA from wild-type cells. However, the precise mechanism of increased *craA* mRNA stability in Cm201 cells is unknown. The generation of stable stem-loop structures within the 5′ UTR mRNA of mutant *bmr3* and *norA* efflux pump transcripts has been suggested as the basis for the observed stability of these transcripts. Similarly, it is tempting to speculate that an analogous mechanism may be responsible for the observed increase in *craA* mRNA stability. However, the relative locations of the *bmr3* and *norA* mutations are significantly more distal in each respective transcript than that described for *craA* here, and as such this mutation may constitute a novel means by which *craA* mRNA stability is enhanced. Alternatively, since the binding of ribosomes to the mRNA transcript has also been shown to have a role in the protection of the transcript from degradation [28], it is a possibility that this mechanism is responsible for the observed increase in *craA* stability. Even though it is not immediately evident how the *craA* mutation may enhance or improve the RBS, it is feasible that the presence of this mutation in the immediate location of the RBS may alter ribosomal binding, thus enhancing *craA* stability.

In summary, we have presented evidence to support the use of the non-pathogenic bacterium *A. baylyi* ADP1 in studies of multidrug efflux. Using this organism, which has a lower level of intrinsic resistance than other strains of *Acinetobacter* associated with nosocomial infections, we have shown how *Acinetobacter spp*. can rapidly adapt to the use of antibacterial chemotherapies *via* the selection of mutant cells which pre-exist in *A. baylyi* ADP1 populations. Remarkably, no less than three different mutations were selected for amongst five different isolates, indicating a high-level of redundancy of multidrug resistance determinants employed by *Acinetobacter spp*., and a propensity for these strains to acquire mutations that lead to drug resistance phenotypes. This rapid and diverse response of *Acinetobacter spp*. to antibiotic challenge may explain the emergence of *Acinetobacter spp*. as an intractable clinical pathogen. Indeed, the increasing importance of multidrug resistant *Acinetobacter spp*. in clinical settings requires novel methods for studies into this growing problem. The genetically malleable organism *A. baylyi* ADP1 may fulfil this developing requirement.

## Materials and Methods

### Bacterial Strains, Media and Reagents

*A. baylyi* ADP1 type strain 33305 was obtained from the American Type Culture Collection (ATCC). *A. baylyi* ADP1 strains were routinely cultured in LB broth supplemented with 5 µg/ml chloramphenicol for single-step mutants or 25 µg/ml kanamycin for allelic replacement derivatives. Culture on solid media was conducted using Nutrient Agar (NA) supplemented with antibiotics at the concentration listed above where required. Bacterial growth assays and MIC analyses were carried out using Mueller-Hinton (MH) broth. Chemicals were purchased from Sigma-Aldrich. Oligonucleotides were obtained from Integrated DNA Technologies, USA, or Sigma-Aldrich, Australia. DNA was amplified in PCR using KOD Hot Start DNA Polymerase (Toyobo Corporation, Japan).

### Bioinformatic Analyses

Efflux proteins encoded by *Acinetobacter spp*. were identified using the Transporter Automated Analysis Pipeline (TransAAP; www.membranetransport.org) [15]. DNA and deduced amino acid sequences were downloaded from relevant *Acinetobacter spp.* genome databases within the National Center for Biotechnology Information (NCBI; blast.ncbi.nlm.nih.gov/). BLAST analyses were undertaken using the suite of online alignment tools available from NCBI. Construction of phylogenetic trees was undertaken using MrBayes software version 3.1.2 based on protein alignments made using ClustalX version 2.0.12. Orthologous efflux systems from different *Acinetobacter* strains were identified by multiway best-match BlastP analyses, using E-value limit of 10^−5^ to designate putative orthologs. Percent identity and similarity were determined using MatGAT (Matrix Global Alignment Tool) v 2.02 [29].

### Generation of mutant *A. baylyi* ADP1 strains

*A. baylyi* single-step mutants were generated by plating 100 µl (2.08×10^8^ CFU) of wild-type *A. baylyi* ADP1 cells from an overnight culture directly onto NA containing either 10 or 20 µg/ml chloramphenicol. Mutant strains were named as per Table 1. For the generation of *A. baylyi* ADP1 allelic replacement strains, a method similar to that of Metzgar *et al.* [2] was employed, using a kanamycin resistance cassette as a selectable marker. Primers used in alleic replacement studies are given in Table 1.

### Bacterial Growth Assays

Wild-type *A. baylyi* ADP1 and single-step mutants were struck for single colonies on NA or NA containing chloramphenicol 5 µg/ml where required. A single colony from each strain was separately inoculated into 5 ml antibiotic-free MH broth and grown with shaking at 37°C for 16 hours. Saturated overnight cultures of *A. baylyi* ADP1 strains were used to inoculate fresh MH broth to an OD_600_ of 0.01 in a volume of 200 µl in a clear plastic flat-bottomed 96-well microplate (Costar, USA). Bacterial growth assays were conducted using a SPECTROstar Nano spectrophotometer (BMG Labtech) equipped with temperature-regulated heating plate at 37°C with shaking in a double-orbital motion. Cell growth was monitored over a time course of 15 hours. Assays were conducted in duplicate using three biological replicates. Doubling times were calculated by plotting the linear regression of the natural log of the optical density against time during exponential growth phase. Differences in growth rates between wild-type and mutant strains were determined using the paired Student's t-test, with a P-value of 0.025 as the cut-off for statistical significance.

### MIC assays

MIC analyses were conducted using the two-fold broth dilution method described by Wiegland *et al.*, 2008 [30]. Briefly, 96-well flat-bottomed microplates were prepared for MIC analyses by inoculating appropriate wells with 200 µl MH broth containing two-fold dilutions of test compounds. *A. baylyi* ADP1 cells grown to an OD_600_ of 0.6 were inoculated into MH containing wells, before microplates were incubated for 16 hours at 37°C with shaking. The presence of bacterial growth in each well was determined at OD_600_ using a SPECTROstar Nano microplate reader (BMG Labtech, USA). A two-fold change in MIC was considered to be a significant indicator of resistance.

### Fluorimetric transport assays

10 mL cultures of wild-type *A. baylyi* ADP1 cells or *A. baylyi* ADP1 single-step mutants were grown without selection to mid-logarithmic phase in LB. Cells were collected by centrifugation and were washed twice with 10 mL 20 mM HEPES buffer (pH 7). Washed cells were resuspended in 10 mL 20 mM HEPES (pH 7) to an OD_600_ of 0.6. 1 mL aliquots of cells were loaded with 15 µM ethidium bromide in the presence of 10 µM of the protonophore CCCP at 37°C for 1 hour. Ethidium bromide loaded cells were collected by centrifugation and washed three times in 20 mM HEPES (pH 7). Cells were reenergised with the addition of 0.4% glucose, and the rate of ethidium bromide export was monitored fluorimetrically using a Perkin Elmer LS 55 fluorescence spectrophotometer over a time course of 600 seconds using excitation and emission wavelengths of 530 nm and 610 nm, respectively.

### qRT-PCR and mRNA stability assays

qRT-PCR was employed as a high-throughput preliminary means of determining the gene/s responsible for antimicrobial efflux in *A. baylyi* ADP single-step mutants. Oligonucleotide primers were designed to target each of the putative efflux pumps identified by TransAAP to be encoded within the *A. baylyi* genome. Nucleotide sequences encoding efflux pumps were downloaded from NCBI, and the online primer design program BatchPrimer3 (v1.0; http://probes.pw.usda.gov/batchprimer3/) was used to aid in oligonucleotide design [31]. Parameters for qRT-PCR oligonucleotide design included: 20 bp length, melting temperature (Tm) of ∼57°C, and amplicon size of ∼200 bp. BatchPrimer3 designed oligonucleotides are listed in Table S1. For qRT-PCR analyses, bacterial cells were grown to mid-logarithmic phase in the absence of antibiotic selection, and total RNA was extracted using the PureLink™ RNA Mini Kit (Invitrogen). Approximately 1 µg of total RNA was reverse-transcribed to cDNA using the SuperScript® VILO™ cDNA Synthesis Kit (Invitrogen). qRT-PCR was conducted in an Eppendorf Mastercycler® ep realplex thermal cycler, using GoTaq™ qPCR Master Mix (Promega). Relative abundance of amplicon products was determined using the comparative C_T_ (cycle threshold; ΔΔC_T_) method [32], normalised against the transcription of the constitutively transcribed *rpoB* gene. Global qRT-PCR analysis was conducted at least once for each efflux pump primer pair, and qRT-PCR for pumps showing significant upregulation (>5 fold; *adeJ* and *craA*) was conducted in quadruplicate.

For *craA* mRNA stability studies, *A. baylyi* ADP1 cultures were grown to mid-logarithmic phase before transcription was arrested with the addition of 200 µg/ml rifampicin. Culture samples were harvested by centrifugation at time points T = 0, 5, 10 and 20 minutes and were immediately snap frozen in liquid nitrogen, prior to storage at −80°C until processing. RNA extraction, cDNA synthesis, and qRT-PCR were conducted essentially as above. The relative abundance of the *craA* transcript relative to the *rpoB* transcript in transcriptionally arrested cells over the time course indicated was calculated using the ΔΔC_T_ method. Experiments were conducted in duplicate using two biological replicates.

## Supporting Information

Dataset S1 **Pairwise percent identity (top right; green) and similarity (bottom left; blue) comparisons of putative drug exporting MFS transporters.** CraA orthologs encoded in *Acinetobacter spp*.,AB57_3600 (AB0057), ABBFA_000365 (AB307-0294), ABAYE0338 (AYE), ACICU_03346 (ACICU), ACIAD3292 (ADP1), A1S_3146 (ATCC 17978) and ABSDF0342 (SDF), were compared to the named previously characterised efflux systems. Sequences were obtained from the Transporter Classification Database (www.tcdb.org).
(XLSX)

Dataset S2 **Pairwise percent identity (top right; green) and similarity (bottom left; blue) comparisons of putative drug exporting RND transporters.** Putative exporters from the RND superfamily encoded in *Acinetobacter* strains AB0057 (locus-tag prefix AB57), AB307-0294 (locus-tag prefix ABBFA), AYE (locus-tag prefix ABAYE), ACICU (locus-tag prefix ACICU), ADP1 (locus-tag prefix ACIAD), ATCC 17978 (locus-tag prefix A1S) and SDF (locus-tag prefix ABSDF) were compared to previously characterised RND superfamily transporters labelled by Genbank protein id. Exporter sequences were obtained from the Transporter Classification Database (www.tcdb.org).
(XLSX)

Table S1 **BatchPrimer3 designed oligonucleotides used in qRT-PCR analyses.**
(DOCX)
