# Supplementary material for: Single-Step Selection of Drug Resistant Acinetobacter baylyi ADP1 Mutants Reveals a Functional Redundancy in the Recruitment of Multidrug Efflux Systems
Source: PLoS One. 2013 Feb 7;8(2):e56090. doi: 10.1371/journal.pone.0056090 (PMC3567077; doi:10.1371/journal.pone.0056090)
Supplement: Table S1 — BatchPrimer3 designed oligonucleotides used in qRT-PCR analyses. (DOCX) [file pone.0056090.s003.docx]

**Supporting Table S1. Oligonucleotide primers used in qRT-PCR.**

| **Locus tag*^a^*** | **Length** | **Tm (ºC)*^b^*** | **GC%*^c^*** | **Product Size*^d^*** | **Sequence** |
| --- | --- | --- | --- | --- | --- |
| ACIAD0228F | 20 | 57.54 | 45 | 201 | ATTGTCAATACCGCACTTCC |
| ACIAD0228R | 20 | 57.52 | 45 | 201 | TAATCCACAACCCAAAGAGG |
| ACIAD0429F | 20 | 57.58 | 45 | 192 | CGAAAGTTTACAGCCCAAAG |
| ACIAD0429R | 20 | 57.34 | 45 | 192 | GGGCCGATACCATACATAAA |
| ACIAD0577F | 20 | 57.58 | 45 | 201 | TATGGATCGTGGCCTAAAAC |
| ACIAD0577R | 20 | 57.58 | 50 | 201 | GTCGGAATCCACTGTCCTAA |
| ACIAD0691F | 20 | 57.46 | 45 | 201 | GATATGGGCCTATTCGATGA |
| ACIAD0691R | 20 | 57.82 | 40 | 201 | GGCTCATGCAAGAACAAAAT |
| ACIAD0756F | 20 | 57.51 | 40 | 198 | GCAATCTGGAACAAACCAAT |
| ACIAD0756R | 20 | 57.57 | 40 | 198 | ACAGGACAATTGCAACAACA |
| ACIAD0783F | 20 | 57.39 | 40 | 200 | CGCTATGATGCGATTATTCA |
| ACIAD0783R | 20 | 57.48 | 45 | 200 | AAAATGACAGGAACCACGAC |
| ACIAD0802F | 20 | 57.49 | 45 | 205 | ATTCAGGCATTAGGTGGATG |
| ACIAD0802R | 20 | 57.51 | 40 | 205 | AGCCAATCAATGCCAATATC |
| ACIAD0927F | 20 | 57.42 | 45 | 202 | ATCTGGCACTTTCTTCGGTA |
| ACIAD0927R | 20 | 57.49 | 40 | 202 | GAAAAACCGCTTTGAATCAG |
| ACIAD1006F | 20 | 57.55 | 35 | 200 | ATCGCTTGGAATTGCTTTTA |
| ACIAD1006R | 20 | 57.52 | 45 | 200 | CCAGTATACCGCCAAACAAT |
| ACIAD1042F | 20 | 57.52 | 45 | 199 | TTTCAGGTCGGTGGAAATAC |
| ACIAD1042R | 20 | 57.51 | 40 | 199 | TGTGCATTCCAACACTGATT |
| ACIAD1158F | 20 | 57.45 | 50 | 201 | AGATGTACCAGGGATTCACG |
| ACIAD1158R | 20 | 57.58 | 45 | 201 | AATGCTTGTCCACCATCTTC |
| ACIAD1159F | 20 | 57.58 | 50 | 196 | GGCTTAAAGATGTGGCTGAG |
| ACIAD1159R | 20 | 57.54 | 45 | 196 | ACCGTAGTCAATTTGGCATC |
| ACIAD1488F | 20 | 57.47 | 45 | 201 | TGTTCGTGAACTGGTGACAT |
| ACIAD1488R | 20 | 57.64 | 55 | 201 | GCAGACTCTCGCATACCTTC |
| ACIAD1514F | 20 | 57.36 | 45 | 205 | ATCCTCTCATGTTGGAATGG |
| ACIAD1514R | 20 | 57.67 | 45 | 205 | CCTTCTGTCGGGTCAATAAA |
| ACIAD1515F | 20 | 57.58 | 45 | 199 | TTATCCCATCCGTTACTTGC |
| ACIAD1515R | 20 | 57.73 | 40 | 199 | AGCAAAACGACAAATCCAAC |
| ACIAD1685F | 20 | 57.82 | 35 | 200 | TTTTAATGCCATTGGGTGAT |
| ACIAD1685R | 20 | 57.51 | 40 | 200 | ATGCTGAATTGGAACCAAAC |
| ACIAD1698F | 20 | 57.46 | 40 | 205 | ATATTGCAGGGCAAAGAGAA |
| ACIAD1698R | 20 | 57.63 | 45 | 205 | TTAATACGTGAGCGTGCTTG |
| ACIAD1773F | 20 | 57.5 | 40 | 202 | TTTTGCACAGCAACGTTTAC |
| ACIAD1773R | 20 | 57.64 | 50 | 202 | CATGGTCAGTGCAGTTGTTC |
| ACIAD1804F | 20 | 57.51 | 50 | 199 | GCAGGTATGGTTGACTTGGT |
| ACIAD1804R | 20 | 57.39 | 40 | 199 | AACAGGCAGAAAGAAATCCA |
| ACIAD1873F | 20 | 57.52 | 40 | 201 | AAAATGTCGGAACCATTCAG |
| ACIAD1873R | 20 | 57.42 | 40 | 201 | GACCAACGACACCAATTTTT |
| ACIAD1971F | 20 | 57.39 | 45 | 201 | ACCGTAAATGGATGGTCTTG |
| ACIAD1971R | 20 | 57.83 | 50 | 201 | CTATTCGGTCACCAAGTGCT |
| ACIAD2122F | 20 | 57.61 | 50 | 203 | GTGCCCTTACCACCAGTTTA |
| ACIAD2122R | 20 | 57.31 | 50 | 203 | TGCAGGCATATACGACAGAC |
| ACIAD2259F | 20 | 57.23 | 40 | 200 | CATTATTGGTGTTCGCTTCA |
| ACIAD2259R | 20 | 57.54 | 45 | 200 | ATGCAGCTCCTTGTTGTTCT |
| ACIAD2385F | 20 | 57.58 | 40 | 194 | GTTTTGGTGGCATGTTTTCT |
| ACIAD2385R | 20 | 57.26 | 50 | 194 | TGCAGAACTGATCAGGACAC |
| ACIAD2404F | 20 | 57.46 | 45 | 201 | CTCACTTTCTTTTCGGTTGG |
| ACIAD2404R | 20 | 57.58 | 45 | 201 | TTGCCCAGAGCTAAAGATGT |
| ACIAD2472F | 20 | 57.46 | 40 | 207 | TTGTAATGGTTCGGGTTTTC |
| ACIAD2472R | 20 | 57.52 | 45 | 207 | TTGTAATGGTTCGGGTTTTC |
| ACIAD2610F | 20 | 57.6 | 45 | 201 | GTGCGTTATGGGCAATCTAT |
| ACIAD2610R | 20 | 57.49 | 45 | 201 | CCAGCCATTCCAATATCAGT |
| ACIAD2650F | 20 | 57.02 | 45 | 200 | CTTGAACCCTTCATGATTCC |
| ACIAD2650R | 20 | 57.58 | 45 | 200 | ATCAGCCAAAACATCCAGAC |
| ACIAD2761F | 20 | 57.45 | 40 | 202 | ATTGGCACTTCCATTGATTC |
| ACIAD2761R | 20 | 57.14 | 45 | 202 | AGAACTGTGTTGTTGCATGG |
| ACIAD2763F | 20 | 57.52 | 40 | 201 | TCTGCTACCTTTGCCATTTT |
| ACIAD2763R | 20 | 57.7 | 55 | 201 | ACCACATACCAGCAGTAGCC |
| ACIAD2836F | 20 | 57.7 | 45 | 200 | ATCGCTTAAACACTGGGATG |
| ACIAD2836R | 20 | 57.67 | 55 | 200 | GTAGGCTCTCTCGCCCTAAT |
| **ACIAD2944F** | 20 | 57.58 | 50 | 201 | GGTCTATCGCTTGAAGAACG |
| **ACIAD2944R** | 20 | 57.7 | 40 | 201 | GTTTGGATCGCCTTTAATGA |
| ACIAD3048F | 20 | 57.55 | 45 | 200 | TCCGATATCGTGATTTCTCC |
| ACIAD3048R | 20 | 57.09 | 40 | 200 | ATTAAACCTGTCGGCAACAT |
| ACIAD3084F | 20 | 57.52 | 35 | 200 | TTTATTTTTCTTCGGCTGGA |
| ACIAD3084R | 20 | 57.32 | 45 | 200 | CCATGCATATGTACCAATCG |
| ACIAD3110F | 20 | 57.37 | 40 | 200 | TTGAAGTTCCAGCCGTTTAT |
| ACIAD3110R | 20 | 57.3 | 50 | 200 | ACCAGTAGGTTCATCGGCT |
| ACIAD3170F | 20 | 57.67 | 50 | 194 | CACCCAACTGGCATCTACTT |
| ACIAD3170R | 20 | 57.52 | 45 | 194 | TTCGGTGAGTAAACCCATTC |
| **ACIAD3292F** | 20 | 57.42 | 45 | 203 | ATTGATGTCTTCCCCAGTTG |
| **ACIAD3292R** | 20 | 57.55 | 40 | 203 | GGATTGCCATTTCCTGTTTA |
| ACIAD3400F | 20 | 57.25 | 45 | 200 | AATGATGAGCTCGGAGATGT |
| ACIAD3400R | 20 | 57.25 | 40 | 200 | TTGGCATGTGTCACCATAAT |
| ACIAD3422F | 20 | 57.49 | 50 | 197 | GCAGGATACTGGGGTAATGA |
| ACIAD3422R | 20 | 57.57 | 40 | 197 | TTCGCTCACTTAACGGTTTT |
| ACIAD3423F | 20 | 57.31 | 35 | 200 | ATGCCATTGTGATGATTGAA |
| ACIAD3423R | 20 | 57.31 | 45 | 200 | AGCCAGTGTAATCGCAAACT |
| ACIAD3450F | 20 | 57.34 | 35 | 199 | ATTTTTGCCTTGCGTATGTT |
| ACIAD3450R | 20 | 57.51 | 50 | 199 | GCAAACCTATCACCACCAGT |
| ACIAD3554F | 20 | 57.51 | 45 | 196 | TTCTGCAAAACCTCTCTGGT |
| ACIAD3554R | 20 | 57.38 | 45 | 196 | ACACAGCGGACTTTCTCAAT |
| ACIAD3617F | 20 | 57.2 | 40 | 199 | CCTTGATCGGATTTTTATCG |
| ACIAD3617R | 20 | 57.57 | 40 | 199 | AAAGCGCCGACATTAATAGA |
| ACIAD3624F | 20 | 57.51 | 50 | 200 | GGTCAGCTTTTTCTCACTGG |
| ACIAD3624R | 20 | 57.46 | 35 | 200 | CCATTTTAATCGCCATCATT |
| ACIAD3678F | 20 | 57.57 | 40 | 200 | ATTTCCGTCGTCATTTATGC |
| ACIAD3678R | 20 | 57.51 | 50 | 200 | ATGGCACCCAACAGACTAAC |
| ***ACIAD0307F*** | 20 | 57.48 | 45 | 200 | CACCTGAAGGTCCAAACATT |
| ***ACIAD0307R*** | 20 | 57.41 | 50 | 200 | CAAGTGACCGTCTGCATCTA |

***^a^***NCBI locus tags for *A. baylyi* genes amplified by qRT-PCR. Primers amplifying upregulated transcripts are shown in bold. Primers corresponding to the *rpoB* housekeeping gene are shown in bold italics.

***^b^***Tm; melting temperature.

*^c^*%GC; percent G+C content of oligonucleotide primer.

*^d^*Expected product size of qRT-PCR amplicon when performed using corresponding oligonucleotide partner.
